# Recent advances in the study of Conbercept for diabetic macular edema

**DOI:** 10.1186/s40942-025-00738-6

**Published:** 2025-10-16

**Authors:** Zhe Liu, Xin Zheng, Hong Li

**Affiliations:** 1 School of Medicine, Northwest Minzu University, Lanzhou, 730050 China; 2 Department of Ophthalmology, The 940th Hospital of Joint Logistics Support Force of Chinese People’s Liberation Army, Lanzhou, 730050 China; 3 Department of Ophthalmology, Yixing People’s Hospital, Yixing, 214200 China

**Keywords:** Anti-VEGF agents, Conbercept, Diabetic macular edema, Diabetic retinopathy, Ophthalmology

## Abstract

Diabetic macular edema (DME) is one of the leading causes of blindness in diabetic retinopathy. As a domestically developed fusion protein drug in China, the anti-vascular endothelial growth factor (VEGF) agent Conbercept has demonstrated significant efficacy in the treatment of DME in recent years. This systematic review explores the mechanism of action and latest research advancements of Conbercept in DME treatment, integrating clinical trial data and comparisons with other anti-VEGF therapies. It further discusses existing limitations in current research and proposes future research directions.

## Background

Diabetes Mellitus (DM) stands as a global public health crisis, with its complications leading to organ damage becoming a primary cause of disability and mortality. According to 2025 data from the International Diabetes Federation (IDF), the global prevalence of diabetes has reached 537 million, projected to exceed 783 million by 2045, with approximately 30% of patients developing diabetic retinopathy (DR) [1]. DME, the most vision-threatening complication of DR, centers on chronic hyperglycemia-induced breakdown of the blood-retinal barrier (BRB), causing vascular leakage in the macular region, abnormal inflammatory factor expression, and eventual fluid accumulation that jeopardizes central vision [2].

Since 2005, anti-VEGF drugs like ranibizumab and aflibercept have become first-line therapies for DME. However, issues such as drug resistance in some patients and the burden of monthly injections leading to decreased compliance persist [3, 4]. In 2011, the World Health Organization’s 51st International Nonproprietary Name (INN) meeting approved the international nonproprietary name “Conbercept” for the active FP3 protein of KH902 and KH903, novel Category 1 biologics from Chengdu Kanghong Pharmaceutical Group. Conbercept was approved in China for wet age-related macular degeneration (wAMD) in 2013 and its indication was extended to DME in 2019, followed by inclusion in China’s national medical insurance. Recent key clinical trials have shown advantages for Conbercept in extending dosing intervals. The 2023 Asia-Pacific Academy of Ophthalmology (APAO) guidelines list it as one of the preferred drugs for DME treatment [5].

## The pharmacological characteristics and mechanisms of action of Conbercept

DME is primarily driven by chronic hyperglycemia, which disrupts retinal homeostasis through multiple pathways: first, it stimulates excessive release of VEGF-A, directly increasing vascular permeability and causing capillary leakage [6]; second, it induces synergistic effects of inflammatory factors such as IL-6 and TNF-α with placental growth factor (PlGF), activating microglial and Müller glial cells (the latter exhibiting abnormal AQP11 expression leading to fluid transport imbalance), thereby exacerbating BRB damage [7, 8]; third, it triggers oxidative stress and accumulation of advanced glycation end-products (AGEs), which disrupt endothelial tight junctions, leading to microaneurysm formation and lipoprotein extravasation, ultimately resulting in macular edema [9]. Conbercept, through its targeted molecular design and multi-targeted action, precisely intervenes in these pathological processes to achieve therapeutic effects for DME, with the following pharmacological mechanisms:

### Molecular structure design

Conbercept, a China-originated recombinant human VEGF receptor-antibody fusion protein, was designed to align with the pathogenic mechanisms of DME. It integrates key functional domains of VEGFR1 (Ig-like domain 2) and VEGFR2 (Ig-like domains 3–4) to specifically target PlGF and VEGF-A associated with DME. Additionally, the fusion of a human IgG1 Fc fragment enhances stability, extending the vitreous half-life to approximately 4.2 days—45% longer than ranibizumab (2.9 days)—and reducing vitreous clearance rates [10, 11]. The 143 kDa molecular weight not only improves binding efficiency for VEGF-A and PlGF but also slows vitreous diffusion, creating a sustained-release effect that supports prolonged blockade of DME’s pathogenic pathways [11].

### Multi-target pharmacokinetics

**Fig. 1:**
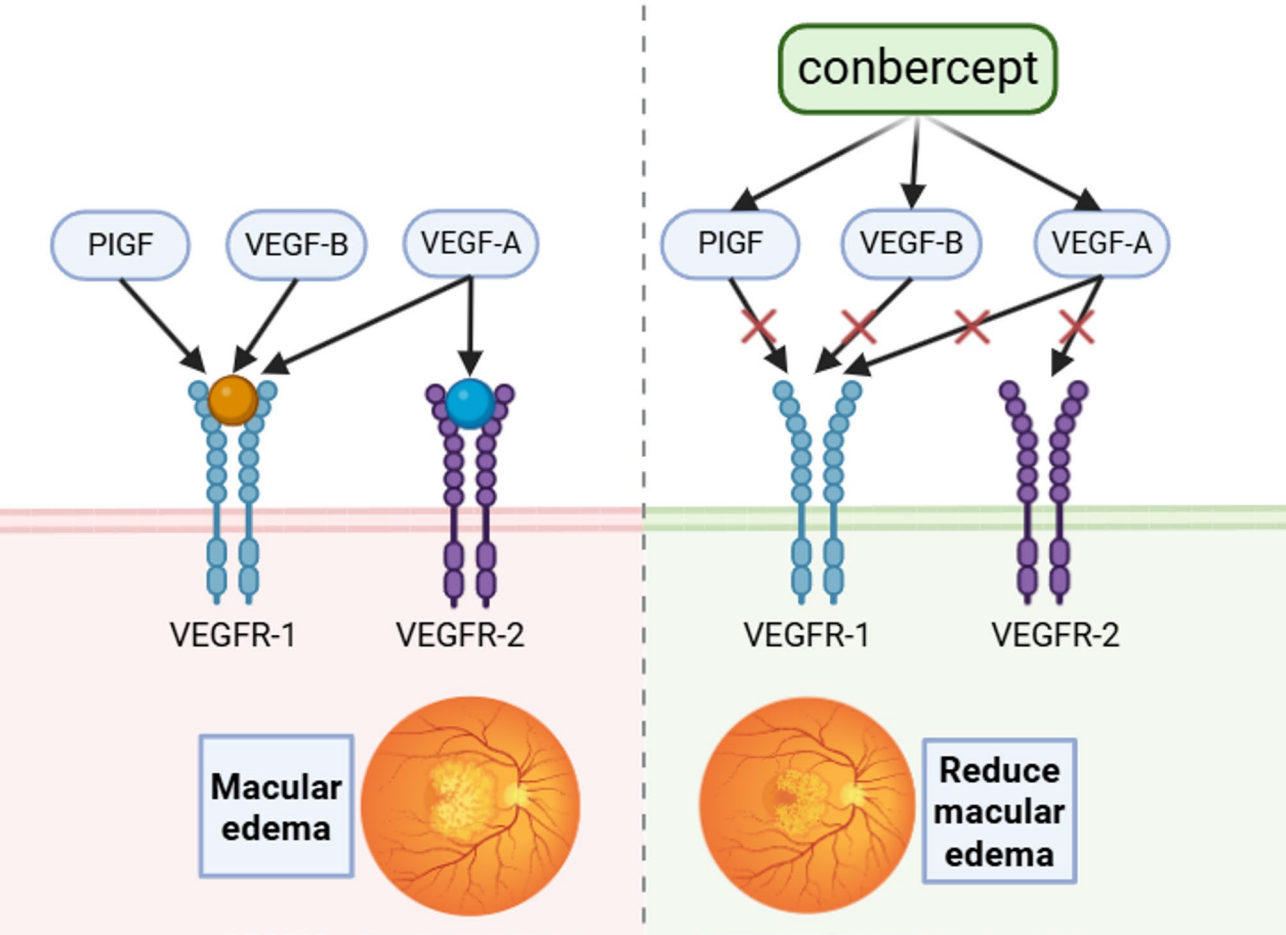
Pharmacological mechanism diagram of Conbercept

Conbercept directly intervenes in pathological processes by competitively binding key DME pathogenic factors. Its specific mechanisms of action are outlined as follows [10]: Table 1, Fig. 1.

**Table 1:** Target-specific actions of conbercept

Target	Biological function	Conbercept intervention mechanism	Clinical significance
VEGF-A	Mediates increased vascular permeability and angiogenesis	Binds and neutralizes free VEGF-A via high-affinity interaction with the VEGFR2 domain	Core therapeutic target; directly inhibits leakage and edema
VEGF-B	Promotes pericyte apoptosis and infiltration of inflammatory cells	Specifically recognizes and binds through the VEGFR1 domain	Reduces chronic inflammation and vascular degenerative changes
PlGF	Activates monocytes/macrophages and participates in immune-inflammatory responses	Forms a “decoy receptor” complex via the VEGFR1 domain, neutralizing PlGF and inhibiting its biological activity	Blocks non-VEGF-A-dependent resistant pathways, improving resistance

### Pharmacokinetic characteristics

The pharmacokinetic profile of conbercept is tailored to meet the long-term treatment requirements for DME: After a single 0.5 mg intravitreal injection, peak concentration (Cmax ≈ 65.62 µg/g) is reached within 6–12 h, with effective therapeutic concentrations sustained for over 34 days, enabling prolonged inhibition of pathogenic factors such as VEGF-A and PlGF that are persistently activated in DME [12]. Additionally, due to its large molecular weight and the localized metabolism characteristics of the intravitreal space (degradation into small peptide fragments via proteolytic enzymes followed by excretion through aqueous humor circulation), systemic absorption is minimal (serum concentration < 0.1 ng/mL). This avoids hepatic and renal metabolic burdens while reducing systemic anti-VEGF-related risks, ensuring safe and sustained intervention targeting the localized pathological pathways in DME [12, 13].

### Mechanism of action

(I) Direct blockade of core pathological pathways in DME

Targeting the key pathways of VEGF-A-mediated vascular leakage and PlGF-synergized BRB disruption in DME, Conbercept’s affinity for VEGF-A is significantly higher than ranibizumab’s, allowing more thorough neutralization of free VEGF-A and rapidly reducing retinal vascular permeability [14]. Simultaneously, through a “decoy receptor” mechanism, it binds PlGF, inhibiting its synergy with inflammatory factors, reducing inflammatory leakage from the BRB, and creating a synergistic blocking effect on core pathogenic factors [15].

(II) Indirect modulation of the retinal pathological microenvironment

Addressing chronic inflammation and microcirculatory disturbances in DME, Conbercept inhibits the VEGF signaling pathway, downregulating levels of inflammatory factors such as ICAM-1, MIP-1, IL-1β, IL-6, and TNF-α. This alleviates overactivation of microglia and Müller cells, reducing inflammation-mediated BRB damage [16]. It also lowers VEGF and ANGPTL-4 levels, improving macular microcirculation and increasing retinal blood supply, indirectly correcting fluid transport imbalances in Müller cells and aiding in the restoration of retinal homeostasis [17].

(III) New mechanism

Recent studies have uncovered a novel mechanism by which Conbercept reduces hard exudates in DR. Clinical observations indicate that three consecutive intravitreal injections of Conbercept significantly decrease the area of hard exudates in DME patients. Mechanistic research reveals that Conbercept inhibits the VEGF/VEGFR2 signaling pathway, reducing oxidative stress and inflammatory responses in retinal Müller cells under hyperglycemic conditions. This further activates the PPARγ-CD36 axis, upregulating the expression of the scavenger receptor CD36, thereby significantly enhancing Müller cells’ phagocytic capacity for oxidized low-density lipoprotein (the primary component of hard exudates) [18].

It should be noted that while Conbercept effectively intervenes in VEGF family factors and inflammation-related pathological pathways in DME, it does not directly address hyperglycemia-induced oxidative stress, AGE accumulation, or endothelial tight junction damage. Clinical management of DME therefore still requires combined glycemic control to achieve comprehensive treatment outcomes.

## Clinical advances in Conbercept treatment for DME

### Efficacy and safety of monotherapy

Multiple clinical studies have confirmed the significant efficacy of Conbercept monotherapy for DME, demonstrating clear superiority over laser therapy in improving visual acuity, anatomical structure, and quality of life. The SAILING study, a multicenter, randomized, double-blind Phase 3 clinical trial, enrolled 251 DME patients randomized into a Conbercept injection group and a retinal laser photocoagulation group. Results showed that at 12 months, the Conbercept group had significant visual acuity improvement, whereas the laser group showed no significant improvement in best-corrected visual acuity (BCVA). The proportion of patients achieving visual acuity gains of ≥ 15, ≥10, and ≥ 5 letters was significantly higher in the Conbercept group at both 6 and 12 months compared to the laser group [19].

Regarding safety, Conbercept has shown a good ocular and systemic safety profile. Ocularly, long-term treatment (24 months) resulted in intraocular pressure elevation in only about 12.8% of patients, with no cases of secondary glaucoma and no significant difference in incidence compared to the laser photocoagulation group [19]. No severe endophthalmitis or vitreous hemorrhage was reported; injection-related adverse events (such as conjunctival hemorrhage) were low in incidence, mild, and transient [20]. Long-term treatment (12 months) observed no new retinal atrophy or fibrosis; OCT showed improved retinal structure without damage [21]. Systemically, studies over 12 months and systematic reviews reported no arterial thromboembolic events (myocardial infarction, stroke) or significant increase in hypertension or thrombotic risk [15]. Even with long-term use in DME patients with comorbid diabetic kidney disease, it did not worsen renal function damage, and relevant laboratory indicators remained stable [22]. For special populations, no cross-adverse reactions were observed 12 months after unilateral injection [23]. In patients with proliferative diabetic retinopathy (PDR) and vitreous hemorrhage, Conbercept showed lower risk of anterior chamber inflammation or good compatibility in combination therapy without increasing complications [24].

### The optimization of treatment protocols

Research on optimizing Conbercept treatment regimens for DME primarily focuses on the core goals of “improving efficacy, reducing burden, and achieving personalized adaptation.”

At the foundational treatment level, the general recommended dose of conbercept is 0.05 mg, equivalent to a 0.05 ml injection volume. Current evidence does not yet support the superiority of alternative dosages (particularly higher doses) in improving DME treatment outcomes. The “3 + PRN” regimen (three consecutive monthly intravitreal injections followed by pro re nata dosing as needed) has been validated for long-term efficacy [20]. Comparative studies between “6 + PRN” and “3 + PRN” regimens demonstrated that the former, by extending the initial intensive treatment phase, significantly improves BCVA and reduces central macular thickness (CMT). Additionally, during the subsequent PRN maintenance phase, the “6 + PRN” approach achieved higher proportions of vision-stable patients and required fewer supplementary injections, offering an optimized option for clinically addressing patients with higher efficacy demands [25].

In the realm of personalized adaptation, generative adversarial networks (GANs) emerged as a critical technical support for analyzing optical coherence tomography (OCT) images. This tool leverages deep learning to extract retinal structural features, enabling predictions of patient outcomes after 52 weeks of Conbercept treatment. Clinicians can utilize these insights to identify distinct response patterns early in therapy, adjust dosing intervals and frequencies tailored to individual needs, and drive the transition from standardized to precision-based treatment paradigms [26].

### The clinical value of combined therapy

Current combined therapy regimens primarily involve anti-VEGF drugs combined with laser or steroid treatments for DME, aiming to complement drug efficacy, improve retinal hemorrhage, and alleviate macular edema. Compared with monotherapy using anti-VEGF agents for persistent DME, the combination of anti-VEGF drugs with intraocular steroids demonstrates significant advantages in improving retinal anatomical structure [27]. However, some studies indicate that beyond two months of treatment, combined therapy is no more effective than monotherapy and is associated with more severe side effects [28].

Studies show that for patients with severe NPDR combined with macular edema, intravitreal injection of Conbercept (IVC) combined with panretinal photocoagulation (PRP) not only effectively improves vision, reduces macular edema, and enhances retinal microcirculation but also significantly lowers aqueous humor levels of SDF-1 and VEGF, with enhanced overall efficacy and good safety [29]. In terms of reducing central macular thickness (CMT) and improving BCVA in DME eyes, the combination of IVC with subthreshold micro-pulse laser (SMLP) is equally effective as IVC alone. Additionally, combined therapy significantly reduces the number of required IVC injections [30]. This suggests that combined therapy can serve as a better adjunctive approach, expanding clinical treatment options.

Currently, some emerging combination therapy regimens are also being actively explored. The combination of Conbercept and calcium dobesilate can better improve the retinal microcirculation status and visual prognosis in DME patients, potentially reducing the need for anti-VEGF injections [31]. While Conbercept has limited direct effects on Ang-2, its future combination with anti-Ang-2 agents (such as Faricimab) could provide more comprehensive target coverage, offering certain benefits for enhancing treatment efficacy [32].

Furthermore, Chinese researchers are actively conducting clinical trials on IVC combined with traditional Chinese medicine (TCM) for DME, exploring the potential of TCM in treating retinal diseases. Preliminary results have been achieved [33], but existing studies use diverse TCM formulas without comparison, and sample sizes are insufficient to validate the long-term efficacy and safety of combined TCM therapy.

### Comparative study of Conbercept and other anti-VEGF agents

Please refer to Table 2 for the comparative details.

**Table 2:** Comparative table of different Anti-VEGF drugs

Comparison Dimension	Conbercept	Ranibizumab	Aflibercept	Brolucizumab/Faricimab
Molecular Properties	VEGFR1/VEGFR2-Fc fusion protein (143 kDa)	VEGF-A antibody fragment (48 kDa)	VEGFR1/VEGFR2-Fc fusion protein (115 kDa)	Brolucizumab: Single-chain antibody fragment (26 kDa) Faricimab: VEGF-A/ANG-2 bispecific (149 kDa)
Target Coverage	VEGF-A/B, PlGF	VEGF-A	VEGF-A/B, PlGF	Brolucizumab: VEGF-A Faricimab: VEGF-A/ANG-2
Binding Affinity (KD)	VEGF-A < sub > 165</sub>: 0.3pM PlGF༚1.8pM	VEGF-A < sub > 165</sub>: 46pM	VEGF-A < sub > 165</sub>: 0.497pM PlGF༚8.5pM	VEGF-A < sub > 165</sub>: 1.0pM(Faricimab)
Key Phase III Trial (BCVA Gain)	SAILING: +8.2 letters(12 months)	Pagoda: +9.4 letters (64 weeks) REFINE: +6.8 letters(12 months)	VIVID/VISTA: +9.4 letters (52 weeks)	Faricimab: YOSEMITE/RHINE: +10.7 letters (1 year)
Maintenance Dosing Frequency	Every 3–4 months (PHOENIX study)	Every 2–4 months (DRCR.net Protocol T)	Every 2–3 months (VIVID/VISTA)	Faricimab: Every 4 months (≥ 60% of patients)
Anatomical Improvement (CST Reduction)	42%	32%	39%	Faricimab: 45%
Long-term Vision Maintenance	2-year results: +8.2 letters	5-year follow-up: +6.5 letters (DRCR.net)	4-year follow-up: +10.2 letters	Data not available
Advantage in Special Populations	Renal impairment patients: BCVA gain + 9 letters	No significant advantage	Moderate advantage	Insufficient data
Pharmacoeconomics (e.g., China)	3452.8 RMB × 6 injections (1st year)	3673.5 RMB × 8 injections (1st year)	4100 RMB × 6 injections (1st year)	Faricimab: 3608 RMB × 3 injections (per year)
Guideline Recommendation Level	APAO 2023: Level I recommendation (Preferred in Asia-Pacific)	NICE 2023: Level II recommendation	AAO 2023: Level I recommendation	Faricimab: FDA Breakthrough Therapy

The tabular data are derived from foundational research and core clinical trials of anti-VEGF agents, such as conbercept: SAILING study, PANDA series studies; aflibercept: VIVID and VISTA studies and their extension studies; ranibizumab: Pagoda study, RIDE and RISE studies and their extension studies, etc [19, 34–43].

The advantages and disadvantages of conbercept in treating DME, as shown in the table above, are as follows:

Advantages:

① Conbercept has broad target coverage, simultaneously targeting VEGF-A/B and PlGF, consistent with aflibercept’s target range and superior to ranibizumab and brolucizumab which only target VEGF-A. Targeting PlGF makes it particularly suitable for patients with inflammation-associated edema. Its affinity for VEGF-A is significantly higher than ranibizumab and slightly better than aflibercept; its affinity for PlGF is also much higher than aflibercept.

② Conbercept shows significant short-term visual acuity improvement and stable long-term maintenance, with excellent anatomical improvement, meaning it can improve patient vision faster and more persistently, resolve macular edema, and more effectively reduce retinal leakage and thickening.

③ Conbercept, confirmed in the PHOENIX study, allows for dosing intervals of every 3–4 months during the maintenance period, offering more flexible injection schedules and reducing patient burden.

④ Clear benefits for special populations (e.g., patients with renal insufficiency).

⑤ Within China’s healthcare economy, Conbercept offers moderate first-year treatment costs and outstanding cost-effectiveness.

Disadvantages:

① Lack of long-term efficacy data. For diabetic patients requiring decades of management, Conbercept lacks longer-term efficacy and safety data support.

② While injection interval flexibility is better than ranibizumab and aflibercept, it is slightly inferior to faricimab.

③ Lacks the dual-target (VEGF-A/ANG-2) synergistic advantage of faricimab. In DME patients with complex vascular pathologies, it may not achieve the synergistic therapeutic effect of “anti-leakage + vessel stabilization”.

④ Conbercept’s molecular weight is larger than brolucizumab. While this doesn’t affect clinical efficacy, it offers no advantage in terms of drug tissue penetration efficiency.

⑤ Currently, Conbercept has less international multicenter clinical evidence and global guideline recognition compared to aflibercept and ranibizumab, which may limit its promotion and application in the international market.

In clinical practice, the most suitable anti-VEGF drug should be selected based on individual patient factors, including disease complexity, comorbidities, treatment duration requirements, and economic considerations.

## Discussion on the limitations of clinical research

### Limitations in study design and sample size

Although existing core clinical trials (such as the SAILING study) employed randomized controlled designs, their sample sizes were mostly limited to 200–500 participants. Additionally, the enrolled populations primarily consisted of patients with moderate baseline visual acuity and no severe comorbidities. This led to insufficient coverage of special DME populations, such as those with severe retinal fibrosis, persistent macular edema (duration exceeding two years), or systemic multi-organ complications (e.g., severe cardiovascular diseases, end-stage renal disease). As a result, the generalizability of the findings to such complex real-world clinical scenarios is limited [19]. Furthermore, most studies had follow-up periods of 12–24 months, lacking long-term efficacy and safety data over five years or more. This makes it difficult to determine the impact of long-term conbercept treatment on retinal neural function and whether delayed ocular adverse events (such as retinal pigment epithelial atrophy) or potential systemic risks (e.g., the effects of long-term VEGF inhibition on vascular repair) exist [20, 21].

### Inadequate research on treatment response variability and drug resistance mechanisms

Clinical studies have observed that approximately 30% of DME patients respond poorly to conbercept treatment [44]. However, current research on drug resistance mechanisms remains superficial. Most existing studies focus on VEGF pathway-related factors (e.g., differences in VEGF subtype expression), while there is limited research on the relationship between non-VEGF-dependent mechanisms (such as oxidative stress and advanced glycation end product accumulation) and drug resistance. Clinically applicable “drug resistance prediction biomarkers” (e.g., serum inflammatory factor levels, genetic polymorphisms) have not yet been established, making it impossible to identify patients at high risk of drug resistance in advance and hindering the implementation of personalized treatment.

### Need for higher evidence level in combination therapy regimens

Current studies on conbercept combination therapies mostly consist of small-sample (≤ 100 participants), single-center prospective trials or retrospective analyses. There is a lack of large-sample, multicenter Phase III randomized controlled studies to validate these findings. For example, although studies combining conbercept with micropulse laser or calcium dobesilate have shown efficacy advantages [30, 31], parameters such as laser energy and frequency, as well as drug dosage and treatment duration, varied across studies. Moreover, the “optimal timing for combination therapy” (e.g., initial combination therapy vs. combination after poor response to monotherapy) remains unclear. These inconsistencies make it difficult to form unified clinical guideline recommendations, leaving clinicians with considerable subjectivity in treatment selection.

### Lack of health economics and accessibility research

Existing studies primarily focus on efficacy and safety, with very few addressing the health economics of conbercept treatment for DME. DME patients require long-term, repeated injections, and the treatment costs (drug expenses + injection procedure fees) significantly impact long-term patient compliance. However, there is a lack of cost-effectiveness analyses for different treatment regimens (e.g., 3 + PRN vs. 5 + PRN), and no accessibility studies have been conducted for different healthcare payment systems (e.g., urban-rural resident medical insurance, commercial insurance). This absence of data hinders evidence-based healthcare policy formulation and cost-effective clinical treatment choices, particularly posing challenges for promotion in primary healthcare institutions.

In summary, addressing the limitations of current research requires conducting larger-sample, longer-follow-up real-world studies, delving deeper into drug resistance mechanisms and establishing prediction models, carrying out high-quality Phase III clinical trials on combination therapies, and supplementing health economics data. These efforts will further enhance the treatment level for DME and improve patients’ visual acuity and quality of life.

## Future research directions

### Expanding the scope of research

The failure of the global Phase III PANDA trial is the primary reason for conbercept’s limited international recognition. A thorough review of the PANDA trial failure should be conducted to analyze the main causes of high participant dropout rates and certain abnormal efficacy outcomes. Carefully restarting rigorous clinical trials with expanded sample sizes, including patients of diverse ethnicities, regions, and comorbidities, will help improve the generalizability of research findings. Studies targeting specific populations should also be carried out to develop effective treatment strategies for a broader patient base.

### Precision medicine and personalized treatment

DME patients exhibit significant individual variability, paving the way for personalized treatment strategies based on genetic testing and biomarker analysis. Through genetic testing, polymorphisms in genes related to drug metabolism (e.g., Conbercept) and drug targets can be analyzed to predict patient response to the medication, enabling the development of customized dosing regimens and treatment durations for different individuals [45]. Additionally, imaging techniques such as OCT and fluorescein fundus angiography (FFA) can be utilized to dynamically monitor macular edema and retinal ischemia, facilitating “as-needed treatment” or “personalized dosing” strategies to avoid both overtreatment and undertreatment [46].

### Innovative drug combinations and administration strategies

IL-6 plays a pivotal role in the inflammatory response of DME. Drugs targeting the IL-6 receptor, such as Tocilizumab, have demonstrated efficacy for refractory DME in clinical trials [47]. PPAR-γ agonists, which improve blood-retinal barrier function through metabolic regulation and anti-inflammatory effects, can be used in combination with Conbercept [48]. This synergistic action may further enhance treatment outcomes for DME, particularly in patients with refractory cases.

The development of novel drug delivery systems is crucial to addressing the burden of frequent intravitreal injections. Long-acting sustained-release formulations, such as biodegradable intravitreal implants, hold promise for significantly reducing injection frequency [49]. Exploring optimized dosing regimens and establishing personalized dosing schedules and frequencies could improve therapeutic efficacy while minimizing unnecessary drug exposure and potential adverse effects.

### Application of new technologies

Leveraging artificial intelligence (AI)-assisted diagnostic technologies, such as analysis of OCT and optical coherence tomography angiography (OCTA) images, enables more precise assessment of retinal lesion severity and drug treatment efficacy, while also detecting subtle lesions and potential risks [50]. Additionally, gene editing technologies offer opportunities to explore therapeutic approaches targeting genes associated with DME pathogenesis, either in combination with Conbercept or as standalone strategies, fundamentally intervening in the disease progression.

## Conclusion

Conbercept, a recombinant VEGF receptor-antibody fusion protein independently developed in China, efficiently blocks VEGF-A/B and PlGF through its multi-target molecular design. Its binding affinity and intravitreal half-life are significantly superior to those of earlier drugs. Basic research has confirmed that conbercept not only directly inhibits vascular leakage and neovascularization but also improves the retinal microenvironment by downregulating inflammatory factors such as ICAM-1, IL-6, and TNF-α. Compared to traditional laser therapy or other anti-VEGF agents, conbercept demonstrates competitiveness in both efficacy and pharmacoeconomic benefits.

However, the lack of large-scale international long-term studies has limited its global recognition. Currently, it is only considered one of the preferred treatments in the Asia-Pacific region. Further exploration is needed to address individual variations in treatment response, the complexity of refractory DME mechanisms, and long-term retinal safety.

Future research should not only focus on conducting large-scale multinational clinical trials involving diverse ethnicities, regions, and comorbidities but also integrate genomics and multimodal imaging (e.g., AI-assisted OCT analysis) to predict treatment efficacy and develop personalized dosing strategies for precision medicine. Innovative drug delivery methods, such as long-acting sustained-release formulations, should be developed to reduce the injection burden and optimize treatment adherence and disease management efficiency.

## Data Availability

No datasets were generated or analysed during the current study.

## Abbreviations

DME: Diabetic macular edema
VEGF: Vascular endothelial growth factor
DM: Diabetes Mellitus
IDF: International Diabetes Federation
DR: Diabetic retinopathy
BRB: Blood-Retinal Barrier
PIGF: Placental Growth Factor
wAMD: Wet age-related macular degeneration
AGE: Advanced glycation end-product
GANs: Generative Adversarial Networks
PRP: Panretinal photocoagulation
IVC: Intravitreal injection of Conbercept
SMPL: Subthreshold micro-pulse laser
CMT: Central macular thickness
BCVA: Best-corrected visual acuity
PRN: Pro re nata
OCT: Optical coherence tomography
OCTA: Optical coherence tomography angiography
FFA: Fluorescein fundus angiography
